# Histology of the whole body of honey bees: tissue fixation and processing

**DOI:** 10.1177/10406387231160767

**Published:** 2023-03-12

**Authors:** Joanie Lussier, Elsa Racine, Marie-Odile Benoit-Biancamano

**Affiliations:** Laboratoire de Santé Animale du Québec, Ministère de l’Agriculture, des Pêcheries et de l’Alimentation du Québec, Saint-Hyacinthe, Québec, Canada; Groupe de recherche sur les maladies infectieuses en production animale (GREMIP), Département de pathologie et microbiologie, Faculté de médecine vétérinaire, Université de Montréal, Saint-Hyacinthe, Québec, Canada; Groupe de recherche sur les maladies infectieuses en production animale (GREMIP), Département de pathologie et microbiologie, Faculté de médecine vétérinaire, Université de Montréal, Saint-Hyacinthe, Québec, Canada

**Keywords:** anatomy, embedding, fixation, histopathology, honey bee

## Abstract

Beekeeping plays a crucial role in biodiversity, pollination, commercial farming, and the worldwide agricultural economy. Histopathology, which is an important tool for the investigation of diseases in vertebrates, is not commonly used in honey bees (*Apis mellifera*). However, histopathology could potentially help the diagnostic investigation of high mortality in bees. We developed a tissue fixation and processing method enabling systematic production of histologic slides adequate for diagnostic and research purposes. Our method uses inexpensive, accessible products and can be realized with conventional pathology laboratory equipment. The quality of histologic slides obtained is similar to those of vertebrate animals processed routinely in pathology laboratories. Histopathology as a diagnostic and research tool will improve the services currently offered to apiarists and could help decrease the mean mortality rate, increase apiarists’ profits, and ensure long-term pollination services.

Depopulation of honey bee (*Apis mellifera*) colonies has enormous economic impact worldwide.^9^ Various hypotheses have been proposed to explain the decline, but systematic study of these potential causes with respect to Koch postulates requires extensive knowledge of the anatomy and biology of the bee as well as the development of specific laboratory tools. To date, the techniques used (bacterial and fungal culture, PCR) are generally the same as those developed for vertebrates.^13^ However, in vertebrates, histopathology plays a central role in disease investigation, especially in farmed animals, in which it can help pinpoint the most likely agent when multiple pathogens are detected or identify a specific disease condition when no agent is found. Unfortunately, histologic study of the whole body is not used commonly for honey bees because the thick cuticle and smooth body hamper conventional fixation and embedding techniques.

Over the years, many attempts have been made to process and embed entomologic specimens. Although most result in adequate quality for basic descriptive goals,^10,14,22^ there is room for improvement in the systematic production of histologic slides adequate for diagnostic purposes. Many of the techniques described suffered from brittleness,^8^ shrinkage,^12,17^ a high level of complexity, or required the use of embedding media, such as resin, which cannot be processed with conventional histotechnology equipment.^1,3,5^ Our aim was to develop a tissue fixation and processing method enabling histologic examination of bee samples under light microscopy, with the ultimate goal of characterizing normal tissue appearance and, eventually, tissue modifications as a consequence of pathogens, intoxication, or other conditions.

Although insects are not covered by the Canadian Council for Animal Care guidelines, we performed all procedures while respecting ethical considerations. Approximately 500 living worker bees were gathered from the entrance of hives (foragers) at the experimental apiary of the Faculté de médecine vétérinaire de l’Université de Montréal (Quebec, Canada). We tested >170 different fixation and processing method combinations (Table 1), technicians assessed qualitatively brittleness and ease of cutting, and pathologists assessed overall slide quality, tissue preservation, and tissue shrinkage. Only the most successful method is described here.

**Table 1.** Fixation methods and softening media tested for preparation of histologic slides from adult honey bees.

Fixation method	Softening medium
*Modified Bouin fluid	*Nair
*Schaffer liquid^12^	Perenyi^2^
Bouin^2,17^	Soft block
Kramer Bouin^2^	Dioxane^2,4,17^
Cal-rite	DMSO^2,14^
Carl^2,11^	EDTA^8,20^
Carnoy^2^	Glutaraldehyde 1–4%^6,15^
Carnoy–Lebrun^2,21^	Diaphanol^7^
Modified Carnoy^2^	
Picro-chloroacetic^2^	
Davidson fixative	
Modified Davidson	
Jeffrey fluid^16^	
Nelson technique^17^	
Zenker acetic	
Modified Jeffrey	
Jeffrey maceration	
Chamberlain	
Schaffner formula	

* The combination of these agents softened the exoskeleton and enabled hardening of soft tissues without making them coalesce or become brittle, while limiting tissue shrinkage.

Bees were euthanized by immersion in 70% ethanol, followed by immediate fixation with Schaffer fixative (1 part 37% formaldehyde to 2 parts 90% ethanol) mixed in a 2:1 ratio with depilatory cream (Nair; Church & Dwight) for 4 h at 4°C. The active ingredients of the depilatory cream are calcium hydroxide and sodium hydroxide. Legs, wings, and antennae were snipped off with scissors to improve sectioning. Stages of subsequent fixation, dehydration, and pre-inclusion in paraffin were done in an automated tissue processor (Tissue-Tek VIP 5; Sakura). Negative pressure was essential within the tissue processor to prevent formation of air pockets beneath the cuticle.

The following procedure was used for specimen preparation:

1. Fixation in modified Bouin solution (12 parts 80% ethanol, 4 parts 37% formaldehyde, 1 part glacial acetic acid) for 14 h at 35°C.
2. Dehydration in ethanol (1 h in 60%, 1 h in 80%, 2 h in 95%, 2 h in 100%) at 35°C.
3. Dehydration in toluene for 2 h at 35°C.
4. Pre-embedding in paraffin for 2 h at 60°C.

The modified Bouin fixative hardens soft tissues without making them coalesce or become brittle, softens the exoskeleton, and causes little change in the dimensions of tissues (limited shrinkage). Puncturing the cuticle with a small (2–3 mm) scissor incision on the abdomen for rapid fixative entry slightly improves the success rate but has no effect on slide quality. The paraffin (Surgipath EM-400; Leica) used contains synthetic polymers that enable sectioning with minimal compression.

Fixed bees were processed and analyzed in the Centre de diagnostic vétérinaire de l’Université de Montréal, in St-Hyacinthe (CDVUM) and Complexe de diagnostic et d’épidémiosurveillance vétérinaires du Québec (CDEVQ). A microtome (355S; Thermo Fisher) was used to produce 4-µm sections. All slides were stained with hematoxylin–phloxine–eosin–saffron.^19^ Slides were examined with an optical microscope, and images were taken with a digital microscope camera (DFC420; Leica).

Many techniques with many variations were tried in efforts to obtain the most satisfactory preparation (Table 1). The initial immersion of bees in 70% alcohol was ideal for rapid euthanasia, and bees generally succumbed within 15 s. The smoothing medium obtained with the depilatory cream gave better results than any other compound described in the literature and was mixed with Schaffer liquid to allow immediate and simultaneous tissue fixation. The fixation medium used during tissue processing (modified Bouin fluid) is accessible, affordable, and offers effective penetration. Bees can be left in Schaffer liquid or modified Bouin fluid for >4 h if there is a delay in processing, although prolonged delay can result in reduced slide quality. The negative pressure provided in the processor markedly improved fixation, hence limiting fixation to 4 h on the countertop and favoring fixation in the processor (14 h) gave the best results. The paraffin, containing synthetic polymers, maintained the original shape of the specimen through subsequent processing. The use of paraffin is preferred to plastic, which is used in numerous studies,^12,17^ because it is less labor-intensive and allows for sectioning of many specimens with regular microtomes available in histotechnology laboratories. The micrographs indicated excellent fixation with no noticeable collapse or shrinkage of the cuticle. Preservation was excellent with no obvious artifacts compared to known morphology (Figs. 1–9).

**Figures 1–3.**
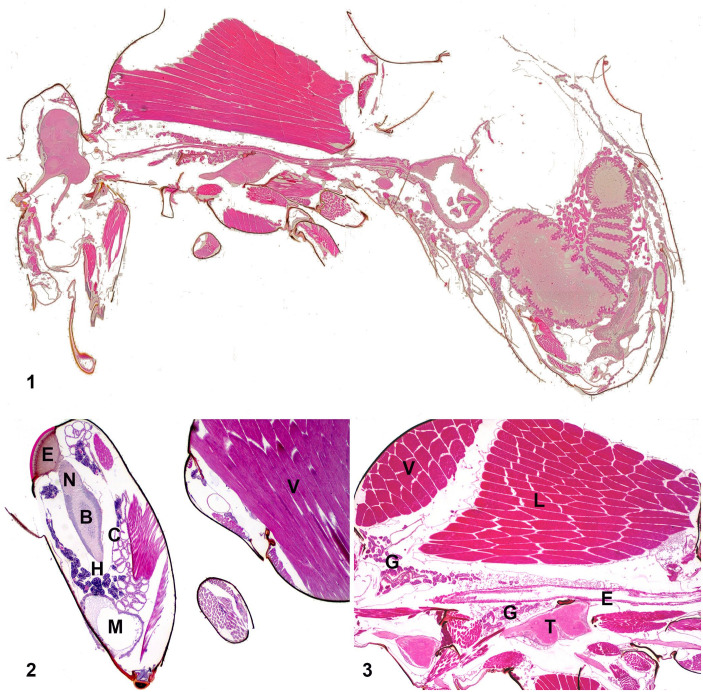
Histologic sections from the whole body of a honey bee worker. **Figure 1.** Whole body, with head to the left. **Figure 2.** Head with hypopharyngeal (H), mandibular (M), and post-cerebral/cephalic salivary (C) glands, compound eye (E), optic nerve (N), and brain (B). Cranial thorax with vertical (V) striated muscles. **Figure 3.** Thorax with longitudinal (L) and vertical (V) flight muscles, esophagus (E), thoracic salivary glands (G), thoracic ganglion (T).

**Figures 4–9.**
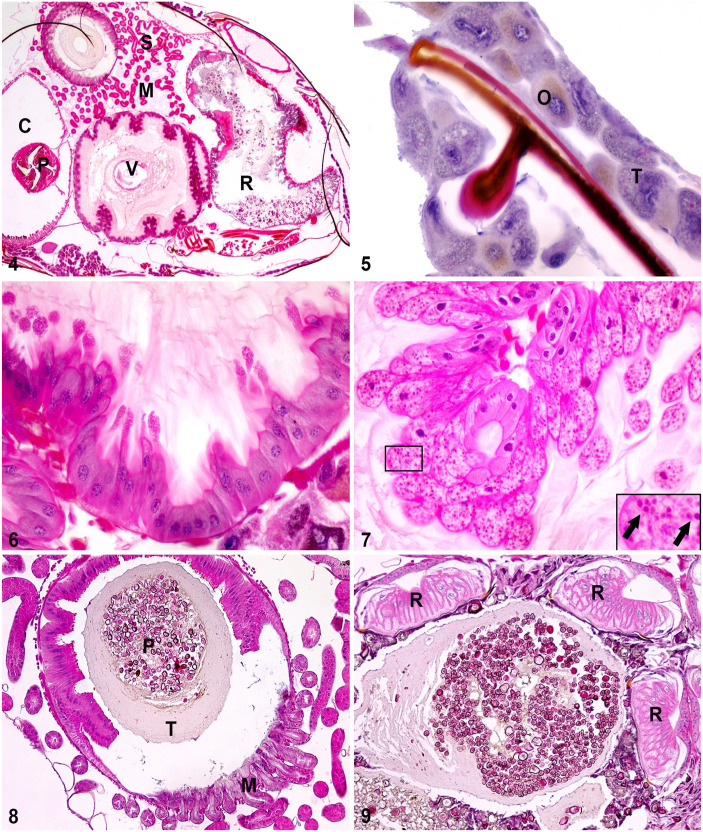
Histologic sections from the whole body of a honey bee worker. **Figure 4.** Abdomen with crop (C), proventriculus (P), ventriculus (V), Malpighian tubules (M), small intestine (S), rectum (R, containing feces). **Figure 5.** Cuticle with trophocytes (T) and oenocytes (O). **Figure 6.** Ventriculus with ciliated epithelium. **Figure 7.** Epithelial cells infected with *Nosema* sp. in a ventriculus. Inset: high magnification of *Nosema* sp. spores (arrows). **Figure 8.** Distal ventriculus and Malpighian tubules (M). Food (P, pollen) is contained within a peritrophic membrane (T). **Figure 9.** Rectum with rectal pads (R).

Our tissue fixation and processing method is simple and reproducible in a conventional pathology laboratory. Histologic slide quality is generally consistent and adequate for histologic characterization and eventual diagnostic purposes. Physiologic variation between same-class individuals (forager worker bees) is thought to be responsible for the rare discrepancies between slides (e.g., slide quality was decreased in wintering bees, possibly as a result of variations in cuticle and body composition, such as increasing fat body). The histologic anatomy of the honey bee has been described in the literature,^22^ and, although published photographic results are of good quality, most of these slides were not initially prepared for a diagnostic purpose. Hence, translation into a laboratory setting can result in quality that is sometimes inferior to the usual mammalian standards. This decline in quality can be explained by the difficult preparation of these specimens (because of the cuticle) and some of the staining methods used (modified Masson).^2^

We share here an adequate tissue fixation and processing method appropriate for histologic slide production; further studies should focus on histologic characterization of known infected populations. Histopathology of honey bee populations can be, as in mammals, a useful diagnostic tool.^18^ Indeed, when combined with other existing diagnostic techniques, such as PCR, the presence of specific histologic lesions could help pinpoint the more likely pathogen in a colony from which multiple agents are isolated. This would hence improve diagnostic services currently offered to apiarists to ensure long-term pollination services.
